# LncRNA PAXIP1‐AS1 fosters the pathogenesis of pulmonary arterial hypertension via ETS1/WIPF1/RhoA axis

**DOI:** 10.1111/jcmm.16761

**Published:** 2021-07-10

**Authors:** Rong Song, Si Lei, Song Yang, Shang‐Jie Wu

**Affiliations:** ^1^ Department of Respiratory Medicine The Second Xiangya Hospital Central South University Changsha China

**Keywords:** ETS1, PAXIP1‐AS1, Pulmonary arterial hypertension, RhoA, WIPF1

## Abstract

Pulmonary arterial hypertension (PAH) is a life‐threatening disease featured with elevated pulmonary vascular resistance and progressive pulmonary vascular remodelling. It has been demonstrated that lncRNA PAXIP1‐AS1 could influence the transcriptome in PAH. However, the exact molecular mechanism of PAXIP1‐AS1 in PAH pathogenesis remains largely unknown. In this study, in vivo rat PAH model was established by monocrotaline (MCT) induction and hypoxia was used to induce in vitro PAH model using human pulmonary artery smooth muscle cells (hPASMCs). Histological examinations including H&E, Masson's trichrome staining and immunohistochemistry were subjected to evaluate the pathological changes of lung tissues. Expression patterns of PAXIP1‐AS1 and RhoA were assessed using qRT‐PCR and Western blotting, respectively. CCK‐8, BrdU assay and immunofluorescence of Ki67 were performed to measure the cell proliferation. Wound healing and transwell assays were employed to evaluate the capacity of cell migration. Dual‐luciferase reporter assay, co‐immunoprecipitation, RIP and CHIP assays were employed to verify the PAXIP1‐AS1/ETS1/WIPF1/RhoA regulatory network. It was found that the expression of PAXIP1‐AS1 and RhoA was remarkably higher in both lung tissues and serum of MCT‐induced PAH rats, as well as in hypoxia‐induced hPASMCs. PAXIP1‐AS1 knockdown remarkably suppressed hypoxia‐induced cell viability and migration of hPASMCs. PAXIP1‐AS1 positively regulated WIPF1 via recruiting transcriptional factor ETS1, of which knockdown reversed PAXIP1‐AS1‐mediated biological functions. Co‐immunoprecipitation validated the WIPF1/RhoA interaction. In vivo experiments further revealed the role of PAXIP1‐AS1 in PAH pathogenesis. In summary, lncRNA PAXIP1‐AS1 promoted cell viability and migration of hPASMCs via ETS1/WIPF1/RhoA, which might provide a potential therapeutic target for PAH treatment.

## 1 INTRODUCTION

Patients with a resting mean pulmonary artery pressure of 25 mm Hg or above are diagnosed as pulmonary hypertension (PH), which is divided into five groups in clinic.^1^, ^2^ Pulmonary arterial hypertension (PAH) is one type of PH, featured with vasoconstriction obstructed pulmonary vasculature, adverse vascular remodelling, vascular fibrosis and stiffening structure. In the past decades, improved understanding of the pathophysiology of PAH leads to the development of efficient clinical drugs targeting vascular remodelling.^3^, ^4^, ^5^, ^6^, ^7^, ^8^, ^9^ For example, the calcium channel blockers, drugs targeting nitric oxide pathway (inhaled NO, PDE5 inhibitors, soluble guanylate cyclase stimulators), endothelin receptor antagonists and drugs targeting the prostacyclin pathway have been reported to ameliorate the pathogenesis of PAH.^10^, ^11^, ^12^, ^13^, ^14^ However, none of these agents is sufficient enough for PAH treatment. Thus, novel drug candidates generated from better understanding of the underlying mechanism of PAH are urgently needed.

In recent years, potential roles of long non‐coding RNA (lncRNAs) have been reported in many diseases, including PAH. For instance, lncRNA TUG1 was found to promote vascular remodelling in PAH.^15^ Zhou et al also showed that polymorphism of MALAT1 contributed to PAH susceptibility in Chinese population.^16^ Moreover, Zahid et al further demonstrated that lncRNAs acted as both promoter and suppressor of pulmonary artery smooth muscle cells’ (PASMCs) proliferation and migration.^17^ Among these lncRNAs, PAXIP1‐AS1 was recently reported to be up‐regulated in PAH and might be associated with the hyperproliferative and migratory actions of IPAH smooth muscle cells.^18^ However, deeper insights for how PAXIP1‐AS1 participates in the pathogenesis of PAH are still urgently needed.

The normal human E26 transformation specific‑1 (ETS1) is a widely accepted proto‑oncogene, which has been found to promote cancer development including breast cancer,^19^ ovarian cancer^20^ and gastric cancer.^21^ Recently, it has been verified that ETS1 also promoted cell proliferation and migration in glioma by regulation of PAXIP1‐AS1.^22^ However, whether such regulatory relationship exists in PAH remains largely unknown.

The small GTPase RhoA and its downstream effectors, the Rho kinases, are considered as important mediators in many cellular processes, such as differentiation, proliferation, survival and migration. Recently, accumulating evidence has presented that RhoA/ROCK pathway plays a vital role in the development and progression of PAH. For example, it has been identified that Rho kinase and RhoA activities were significantly increased in lung tissues of patients diagnosed with idiopathic PAH.^12^ The activity of RhoA/ROCK pathway is also closely associated with PAH development.^23^ Additionally, RhoA activation and ROCK levels were reported to be elevated in chronic hypoxic lungs.^24^ Besides, in a recent research, RhoA was found as a downstream target of WIPF1, an oncogene in many cancers.^25^ Interestingly, WIPF1 could be regulated by ETS1 in lung cancer.^26^ However, the underlying mechanisms of relationship among ETS1, WIPF1 and RohA in PAH have not been noticed yet.

Thus, we aimed to investigate the role of PAXIP1‐AS1 and its relationship with ETS1/WIPF1/RohA signalling in PAH and explore the downstream potential mechanisms participating in the regulation of PAH pathological process. Our study validated that PAXIP1‐AS1 might be a therapeutic target for PAH treatment.

## 2 MATERIALS AND METHODS

### 2.1 Animal studies

A total of 60 adult male Sprague‐Dawley rats (200 ± 20 g, 4‐5 wk) obtained from Bioray Lab (Bioray, Shanghai, China) were kept in a pathogen‐free environment with a 12‐hr light/dark cycle with a temperature of 22 ± 1˚C and a humidity of 40%–60%. Animals were fed with a standard laboratory chow and water ad libitum for the duration of the experiments. The rats were randomly divided into normal group and PAH group. For induction of PAH, the rats were given by a subcutaneous injection of monocrotaline (MCT, Sigma Chemicals, St. Louis, MO, USA; 60 mg/kg), while the normal group was given a single injection of saline as a control. For treatment of PAH, animals injected with MCT were orally given fasudil (100 mg/kg per day) from the day given MCT injection. For inhibition of PAXIP1‐AS1, tail vein injection was conducted with lentiviral plasmid that stably expressing sh‐RNAs targeting PAXIP1‐AS1 or negative control (lent‐sh‐PAXIP1‐AS1/NC, active titre 2*10^8^ TU/mL, GenePharma, Shanghai, China). The rats in all the groups were killed after intraperitoneal injection with 10% chloral hydrate, and samples of lung tissues were removed from each rat and were divided into two groups. One group was flash‐frozen in liquid nitrogen and stored at ‑80˚C for Western blot analysis. The remaining tissues were fixed in 4% paraformaldehyde buffer for histopathological examinations. All animal care and experimental procedures were approved by the Animal Care Ethics and Use Committee of the Second Xiangya Hospital of Central South University and were performed in accordance with the guidelines of this Committee.

### 2.2 Right ventricle system pressure (RVSP) measurements

After the animals were anaesthetized by inhalation of a mixture of isoflurane (4%) and oxygen, the polyethylene catheters were inserted into the right ventricle for haemodynamic measurements. A polygraph system (AP‐601G, Nihon Kohden) was used to measure the right ventricle systolic pressure and systemic blood pressure.

### 2.3 Histological examination

Paraffin‐fixed lung tissues were cut into 4 μM sections using a rotary microtome (Leica, Mannheim, Germany) and were then subjected to HE staining. The orientation of collagen fibres was examined with Masson trichrome staining according to the manufacturer's instruction. For immunohistochemistry staining, slides were stained with antibodies against RhoA (ab54835, Abcam, Cambridge, USA; 1:100) and ɑ‐SMA (ab124964, Abcam, Cambridge, USA; 1:100). The tissues were analysed using a microscope (DP73; Olympus Corporation, Tokyo, Japan).

### 2.4 Cell culture

Human pulmonary artery smooth muscle cells (hPASMCs), purchased from Cascade Biologics Inc (Portland, Oregon, USA), were cultured with SmGM‐2 BulletKit media (Lonza Japan, Tokyo, Japan) supplemented with 5% foetal bovine serum (FBS; Hyclone, Logan, UT, USA), 100 U/ml penicillin and 100 μg/ml streptomycin in a humidified incubator with 5% CO_2_ at 37˚C. For hypoxia environment, hPASMCs were exposed to 1% O_2_ for indicated times.

### 2.5 Cell transfection

The expression plasmid was constructed as previously reported.^27^ Briefly, RhoA, WIPF1 or PAXIP1‐AS1 (GenBank Accession NM_001664.2) cDNA was amplified by PCR and then cloned into the pcDNA3.1( + ) vectors (Invitrogen, Carlsbad, CA, USA). cDNA fragments were amplified by PCR with the use of human wild‐type RhoA, WIPF1 or PAXIP1‐AS1, respectively. Then, cDNAs were inserted into the pcDNA3.1( + ) vectors. sh‐RNAs targeting PAXIP1‐AS1, ETS1 or WIPF1 were purchased from GenePharma, Shanghai, China. Plasmids (5 nM), sh‐RNAs (5 nM) or negative controls (NC) (5 nM) were transfected into PASMCs with Lipofectamine 2000 reagent (Invitrogen, Carlsbad, CA, USA) according to the manufacturer's protocols. After 48 h of transfection, cells were collected for further experiments.

### 2.6 CCK‐8 assay

For measurement of cell viability, cells were seeded at density of 2.5 × 10^5^ in 96‐well culture plates and were cultured overnight. After transfection for 8 h, cells were further cultured and cell viability was measured at 24, 48 and 72 h. Then, 10 μl of CCK‐8 solution was added to each well and cells were incubated at 37˚C for another 1 h. The absorbance of the solutions was detected at 490 nm by a SYNERGY‐HT multiwell plate reader (Synergy HT, Winooski, VT, USA).

### 2.7 BrdU incorporation assay

The cell proliferation was determined using BrdU assay following the manufacturer's instructions. Briefly, the cells were grown on coverslips (Thermo Fisher Scientific) and then incubated with BrdU (20 μM) for 4 h. Cells were then permeabilized with 0.1% Triton X‐100 in PBS and blocked with 3% FBS solution. Cellular DNA was denatured using DNasel treatment. The incorporated BrdU was stained with Alexa Fluor 647 anti‐BrdU monoclonal antibody (BD Biosciences, USA). The nuclei were counter‐stained with DAPI (Sigma). Images were acquired using a Carl Zeiss fluorescence microscope.

### 2.8 Immunofluorescence of Ki67

For Ki67 staining, cells were fixed in ice‐cold methanol for 15 min and allowed to dry for 20 min. Subsequently, cells were incubated with primary antibodies against Ki67 (Cell Signaling Technology, Danvers, MA, USA) at 4˚C overnight after being blocked, followed by a secondary antibody incubation. Nuclei were visualized by counterstaining the cells with DAPI (Invitrogen). Fluorescence intensity was recorded using a fluorescence microscope IX83 (Olympus).

### 2.9 Wound healing assay

Cell migration ability of hPASMCs was tested using the scratch wound assay and then seeded into 6‐well plates for 24 h. Cell layers were scratched using a 200 μl pipette tip to form wound gaps, and the cells were maintained in DMEM with 10% FBS. The cells were photographed at 0 and 24 h to record the wound width.

### 2.10 Transwell assay of cell migration

The migratory capability was detected using transwell chambers (8 μm pores, Millipore, USA). Briefly, cell were digested, resuspended and then plated on the surface of upper chambers. The media containing 10% FBS was injected into the lower chambers to stimulate cell migration. After 8‐h incubation, membranes at the subjacent sides of upper chambers were fixed with 4% PFA and then stained with 0.1% crystal violet stain solution (Solarbio, China). Images were taken by an optical microscope (Leica DM3000, Germany).

### 2.11 Protein extraction and Western blotting

Protein was extracted using RIPA, and the concentration was determined by BCA assay. Equal aliquots of 15 μg total proteins were separated by SDS‐PAGE. Then, proteins were transferred to a PVDF membrane (EMD Millipore, Billerica, MA, USA), then blocked with 5% bovine serum albumin in TBST and incubated in primary antibodies overnight at 4˚C followed by incubation of secondary antibodies for 1 h at 37˚C. Primary antibodies listed were as follows: antibodies against RhoA (#2117, Cell Signaling Technologies, Boston, MA, USA; 1:1000), ROCK1 (#4035, Cell Signaling Technologies, 1:1000), β‐actin (#4970, Cell Signaling Technologies, 1:1000), WIPF1 (ab132512, Abcam, 1:1000), ETS1 antibodies (ab63302, Abcam, 1:1000) and PCNA (Sigma‐Aldrich, USA). And the horseradish peroxidase–conjugated secondary antibodies against rabbit‐IgG and mouse‐IgG (diluted for 1:2000) were purchased from Santa Cruz Biotechnology (Heidelberg, Germany). Protein bands were then visualized using the enhanced chemiluminescence detection system (Bio‐Rad Laboratories, Mississauga, Canada). The intensity of the bands was quantified using ImageJ software tools.

### 2.12 RNA extraction, reverse transcription and quantitative PCR (qRT‐PCR)

The expression of WIPF1, RhoA and ETS1 was determined by qRT‐PCR. Total RNA was extracted with TRIzol reagent (Invitrogen, Carlsbad, CAUSA) according to the manufacturer's instructions, and each sample was reversely transcribed to complementary DNA (cDNA) using the PrimeScript® RT Reagent Kit with gDNA Eraser (Takara, Dalian, China). Quantitative PCR was employed to measure the relative gene expression using the SYBR® Premix Ex TaqII kit (Takara). The reactions were performed in triplicate for each cDNA on the Applied Biosystems Step One Plus Real‐Time PCR System (Applied Biosystems, Foster city, CA, USA). The relative expressions were calculated by 2^‐△△Ct^. β‐actin was used as the internal control. Primers used in this study are shown in **Table** **1**.

**TABLE 1:** Primers used in this study

PAXIP1‐AS1	Forward: 5’‐GAAGTTGGGAGAAGAAAT‐3’
	Reverse: 5’‐AGTGTACCGCAGAGTAAT‐3’
GAPDH	Forward: 5’‐CTGACTTCAACAGCGACACC‐3’
	Reverse: 5’‐GTGGTCCAGGGGTCTTACTC‐3’
RhoA	Forward: 5’‐ATTCGTTGCCTGAGCAATGG‐3’
	Reverse: 5’‐TGTGTCCCACAAAGCCAACT‐3’
WIPF1	Forward: 5’‐AATGGTGCCTTACTTTGTGATT‐3’
	Reverse: 5’‐TTTCTTCCTCTACGGTCCTTG‐3’

### 2.13 RNA immunoprecipitation (RIP) assay

RIP was performed using Magna RNA‐binding protein immunoprecipitation kit (Millipore Corp., Bedford, MA, USA). The cells were lysed in complete RNA lysis buffer and incubated in RIP immunoprecipitation buffer containing magnetic beads conjugated with anti‐ETS1 antibody (diluted for 1:200, Abcam, Cambridge, USA) or IgG control (diluted for 1:100, Abcam). The immunoprecipitated RNAs were then isolated by Proteinase K and subjected to subsequent qRT‐PCR detection.

### 2.14 Chromatin immunoprecipitation (ChIP) assay

ChIP assay was performed using Pierce Agarose ChIP kit (Pierce) according to the manufacturer's instructions. Briefly, cells were transfected with sh‐NC or sh‐PAXIP1‐AS1, respectively. Cells were cross‐linked in 1% formaldehyde and lysed at 48 h post‐transfection. Chromatin was digested using micronuclease. Sheared DNA was incubated with anti‐WIPF1 antibody. Normal IgG was used as a negative control. DNA was purified and analysed by PCR.

### 2.15 Co‐immunoprecipitation (Co‐IP)

Cell extracts were incubated with antibodies against WIPF1 or RhoA (Cell Signaling Technologies, Boston, MA, USA) followed by addition of protein A/G‐Sepharose overnight at 4˚C with constant head‐to‐tail rotations. Sepharose beads were pre‐blocked with 2% BSA in PBS before being used for immunoprecipitation. After incubation, proteins bound to Sepharose beads were washed twice with the lysis buffer, four times with ice‐cold PBS and mixed with 2x SDS‐PAGE gel loading buffer and boiled for 5 min. Then, the supernatant (20 μl per lane) was loaded onto a gel for SDS‐PAGE for electrophoresis and subsequent immunoblot was analysed with the specific antibodies.

### 2.16 Luciferase reporter assay

The dual‐luciferase reporter assay was utilized to determine the interaction of ETS1 and WIPF1 and was conducted according to the method described by Zhang et al.^28^ Briefly, to obtain the mutant WIPF1 3’UTR (3’UTR‐MUT), the conserved binding sites for ETS1 were mutated by one‐step overlap extension PCR. The fragments including the 3’UTR‐WT (Wildtype) or 3’UTR‐MUT regions of WIPF1 were inserted into XhoI/NotI‐digested psiCHECK‐2 vector (Promega) with a renilla and firefly luciferase reporter gene. Then, the psiCHECK‐2 vectors with 3’UTR‐WT or 3’UTR‐MUT regions of WIPF1 and ETS1 plasmids were cotransfected into PASMCs. Forty‐eight h after transfection, cells were collected, and the firefly and renilla luciferase activities were detected by using a dual‐luciferase reporter assay system (Promega) according to the manufacturer's instructions.

### 2.17 Statistical analysis

The data were presented as mean ± standard deviation (SD) from three independent experiments. For comparisons between two groups, Student's *t* test was employed and comparisons between groups were analysed by using one‐way ANOVA. Pearson analysis was used to analyse the correlation between factors. A two‐sided value of *P* < .05 was considered statistically significant.

## 3 RESULTS

### 3.1 Expression patterns of PAXIP1‐AS1 and RhoA were increased in MCT‐induced rat lung tissues

**FIGURE 1:**
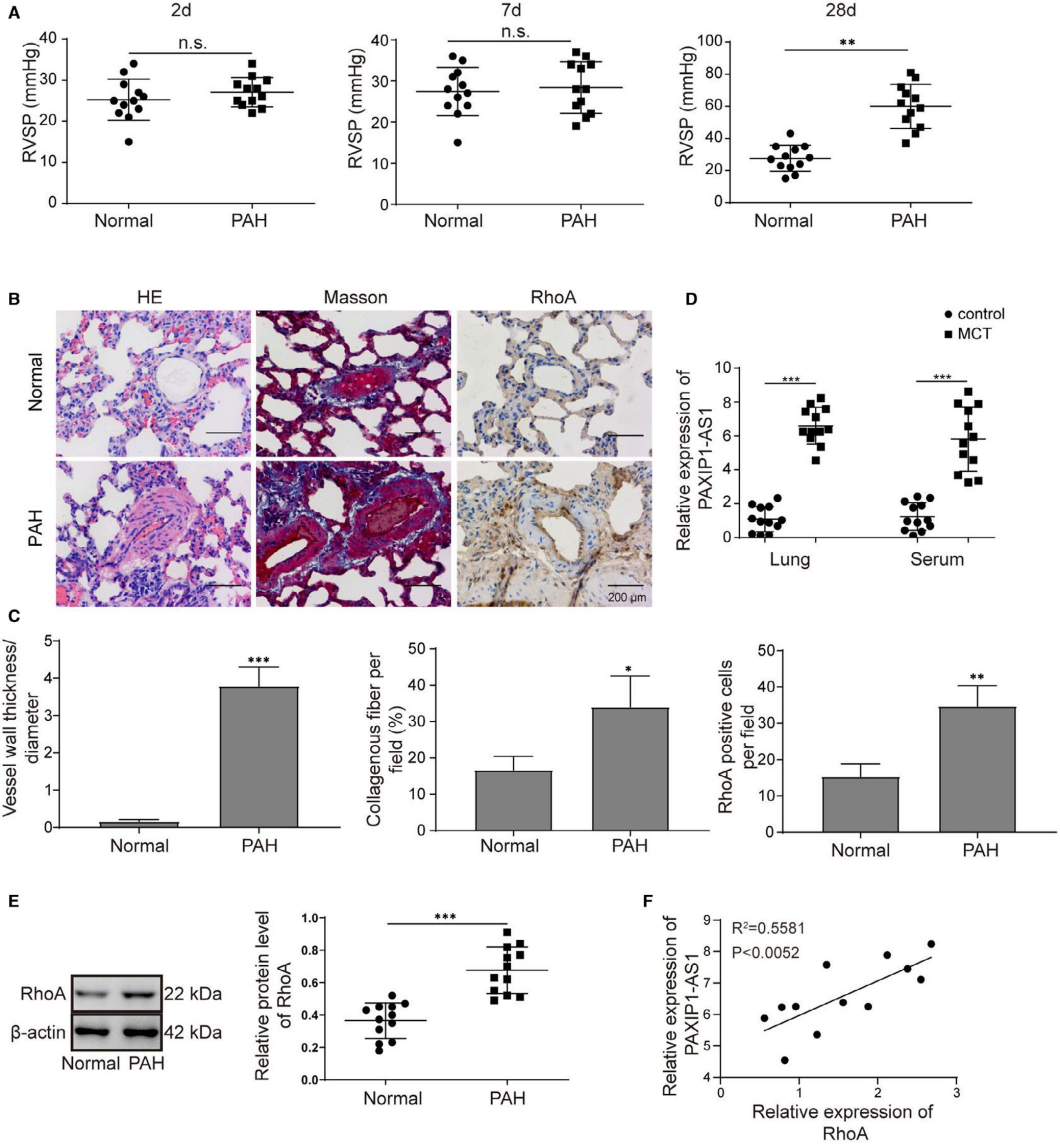
Expression patterns of PAXIP1‐AS1 and RhoA in MCT‐induced rat lung tissues. (A) Right ventricle system pressure (RVSP) of healthy rats and MCT‐induced PAH rat models. (B and C) H&E staining Masson's trichrome staining and IHC for RhoA of lung tissues. Scale bar, 200 μm. (D) Expression levels of PAXIP1‐AS1 in lung tissues and serum from healthy rats or MCT‐induced PAH rat models determined by qRT‐PCR. (E) Expression levels of RhoA in lung tissues from healthy rats or MCT‐induced PAH rat models determined using Western blotting. (F) Pearson correlation analysis was performed to determine the relation between RhoA and PAXIP1‐AS1 in MCT‐induced PAH rat models. Data are presented as the means ± SD of three independent experiments (n = 3). *P < .05, **P < .01 and ***P < .001

To investigate the role of PAXIP1‐AS1 in PAH, in vivo rat PAH model was established by MCT induction. Firstly, RVSP in PAH rat was significantly elevated from 28 days after MCT injection (**Figure** **1A**). Besides, as shown in **Figure** **1B,C**, histological examinations of H&E and Masson staining also displayed significant thickening of vessel walls, narrowing of vascular cavities and dramatic collagen deposition in lung tissues of MCT‐induced PAH rat model group compared with the control. Immunohistochemical (IHC) detection further showed higher expression levels of RhoA in lung tissues of MCT‐induced PAH rat model. Moreover, the expression of PAXIP1‐AS1 was remarkably higher in both lung tissues and serum of MCT‐induced PAH rats than the control rats (**Figure** **1D**). Besides, the protein levels of RhoA were also significantly up‐regulated in MCT‐induced PAH model (**Figure** **1E**). Further, Pearson correlation analysis also revealed the positive correlation of RhoA and PAXIP1‐AS1 in MCT‐induced rat lung tissues (**Figure** **1F**). Taken together, RhoA and PAXIP1‐AS1 were increased in MCT‐induced rat lung tissues, indicating their biological functions in PAH progression.

### 3.2 Knockdown of PAXIP1‐AS1 suppressed hypoxia‐induced cell viability and migration of hPASMCs

**FIGURE 2:**
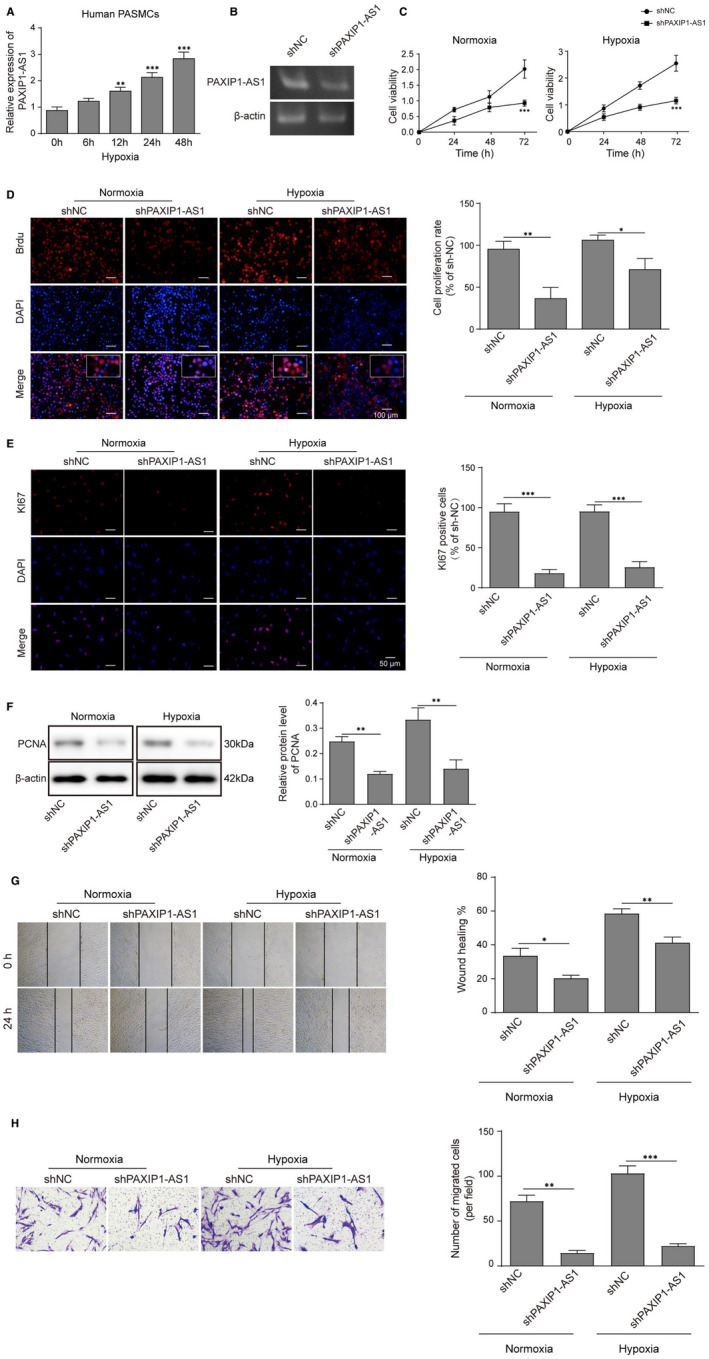
Knockdown of PAXIP1‐AS1 suppressed hypoxia‐induced cell viability and migration of hPASMCs. (A) Expression of PAXIP1‐AS1 under hypoxia condition in hPASMCs. (B) Transfection efficacy of knockdown of PAXIP1‐AS1 by qRT‐PCR. Cell viability was determined by CCK‐8 assay (C), BrdU incorporation assay (D) and immunofluorescence of Ki67. (F) Western blotting analysis of PNCA expression levels. (G and H) Cell migration was measured by wound healing and transwell assays. Data are presented as the means ± SD of three independent experiments (n = 3). *P < .05, **P < .01 and ***P < .001

To further validate the exact role of PAXIP1‐AS1 in PAH pathogenesis, hPASMCs were induced under hypoxia condition to establish in vitro model. It was observed that the expression of PAXIP1‐AS1 was gradually increased following the hypoxia treatment (**Figure** **2A**). As expected, hPASMCs transfected with sh‐PAXIP1‐AS1 displayed the decreased expression levels of PAXIP1‐AS1 (**Figure** **2B**). CCK‐8 assay showed that the increased cell viability of hPASMCs induced by hypoxia treatment was significantly impaired by inhibition of PAXIP1‐AS1 (**Figure** **2C**). Similarly, BrdU incorporation assay was employed to assess the cell proliferation and the data implied that knockdown of PAXIP1‐AS1 also significantly reduced the amount of BrdU‐positive cells (**Figure** **2D**), which was also validated using immunofluorescence of Ki67 (**Figure** **2E**) In addition, Western blot analysis of proliferation marker, PCNA, also presented a significant decrease within PAXIP1‐AS1 knockdown (**Figure** **2F**). Additionally, wound healing and transwell assays demonstrated that hypoxia markedly enhanced the migration ability of hPASMCs, while suppression of PAXIP1‐AS1 dramatically decreased the effects (**Figure** **2G,H**). All these results suggested that knockdown of PAXIP1‐AS1 suppressed cell viability and migration of hPASMCs.

### 3.3 PAXIP1‐AS1 positively regulated the expression of WIPF1 through assembling ETS1

**FIGURE 3:**
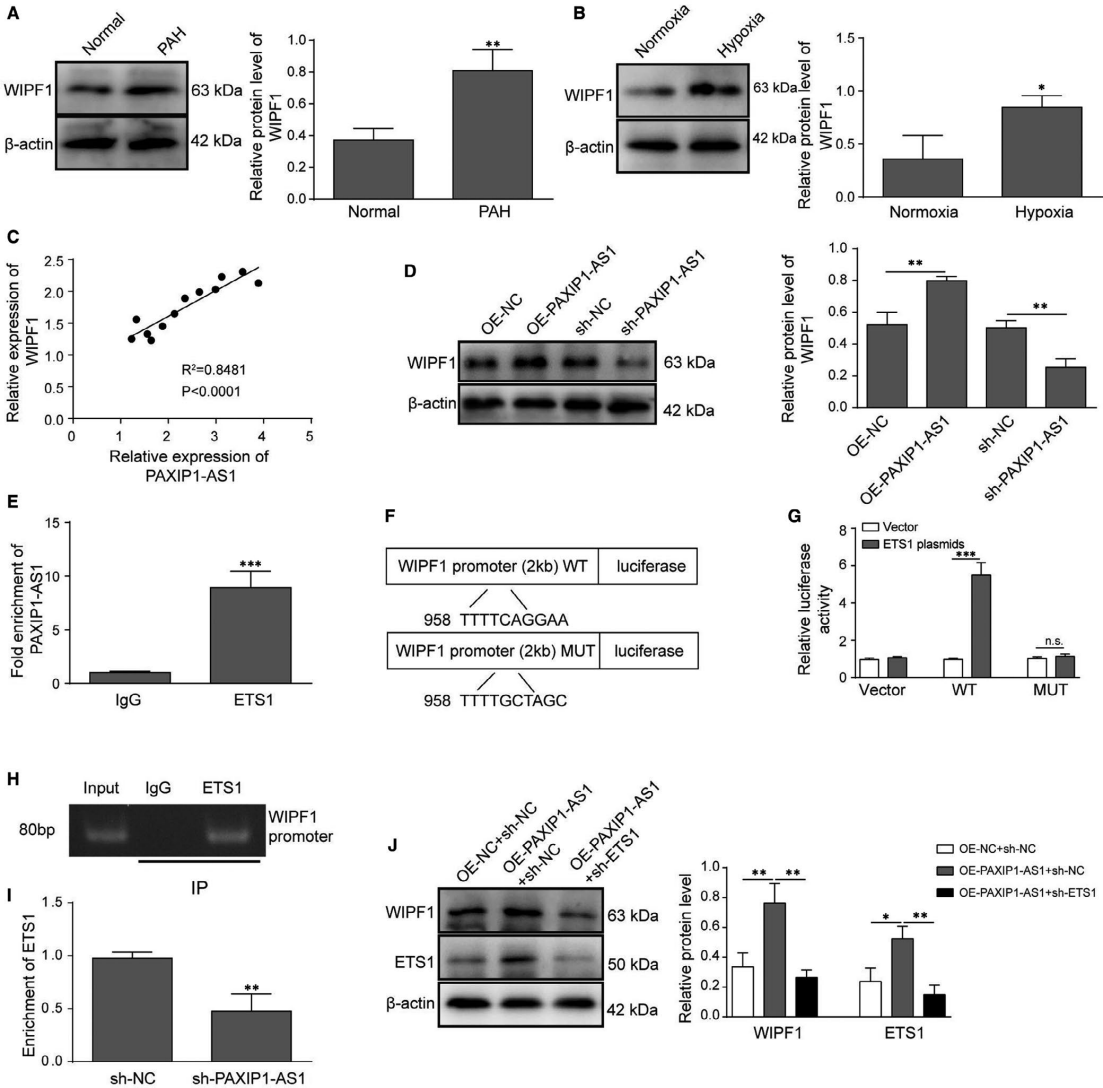
PAXIP1‐AS1 positively regulated the expression of WIPF1 through assembling ETS1. (A) Protein levels of WIPF1 were determined in MCT‐induced rat lung tissues by Western blotting. (B) Western blotting analysis was performed to measure the protein levels of WIPF1 in hypoxia‐induced hPASMCs. (C) Pearson correlation analysis was conducted for correlation between PAXIP1‐AS1 and WIPF1. (D) Protein levels of WIPF1 were determined in hypoxia‐induced hPASMCs transfected with oe‐PAXIP1‐AS1 or sh‐PAXIP1‐AS1. (E) RIP assay was conducted for examining the interaction between PAXIP1‐AS1 and ETS1. (F) Prediction binding mode between ETS1 and WIPF1. (G) Dual‐luciferase reporter assay was conducted for interaction between ETS1 and WIPF1. (H) CHIP assay was subjected to the binding relationship between ETS1 and WIPF1 promoter. (I) CHIP was employed for the interaction between ETS1 and WIPF1 promoter when PAXIP1‐AS1 was knocked down. (J) Protein levels of WIPF1 and ETS1 were determined using Western blotting. Data are presented as the means ± SD of three independent experiments (n = 3). *P < .05, **P < .01 and ***P < .001

To further investigate the underlying molecular mechanisms of PAXIP1‐AS1 in PAH, the relationship between PAXIP1‐AS1 and ETS1/ WIPF1 was studied. Firstly, the expression of WIPF1 was determined in both in vivo and in vitro PAH models and the results showed that the expression of WIPF1 was remarkably elevated compared with the control in both in vivo and in vitro PAH models (**Figure** **3A,B**). Pearson correlation analysis also revealed that expression of WIPF1 was positively correlated with PAXIP1‐AS1 in MCT‐induced rat lung tissues (**Figure** **3C**). Moreover, the data of Western blot analysis determined the positive regulation of PAXIP1‐AS1 on WIPF1 expression. As expected, PAXIP1‐AS1 overexpression significantly enhanced the expression of WIPF1, while the inhibition of PAXIP1‐AS1 led to the opposite result (**Figure** **3D**).

The data of gene co‐expression network using Coexpedia database showed that WIPF1 ranked in the first place of the potential genes co‐expressed with ETS1.^26^ We further found that ETS1 has a binding region in the promoter of WIPF1 with JARSPAR database. To further confirm the regulatory effects of PAXIP1‐AS1 on WIPF1, RIP assay was conducted and the data verified the direct binding relationship between PAXIP1‐AS1 and ETS1 (**Figure** **3E**). Besides, dual‐luciferase reporter assay also showed that the luciferase activity was remarkably higher when ETS1 was overexpressed in WT‐WIPF1; however, no significant difference was found in MUT‐WIPF1 (**Figure** **3F,G**). Additionally, ChIP analysis demonstrated that ETS1 directly bound to the promoter region of WIPF1 and the enrichment of ETS1 markedly decreased when PAXIP1‐AS1 was suppressed (**Figure** **3H,I**). Further, Western blotting assay further validated that overexpression of PAXIP1‐AS1 led to significant up‐regulation of ETS1 and WIPF1, while these effects were abolished by silencing ETS1 (**Figure** **3J**). All these findings suggested that PAXIP1‐AS1 positively regulated the expression of WIPF1 through assembling ETS1 protein.

### 3.4 PAXIP1‐AS1 promoted cell viability and migration of hPASMCs through ETS1/WIPF1 signalling

**FIGURE 4:**
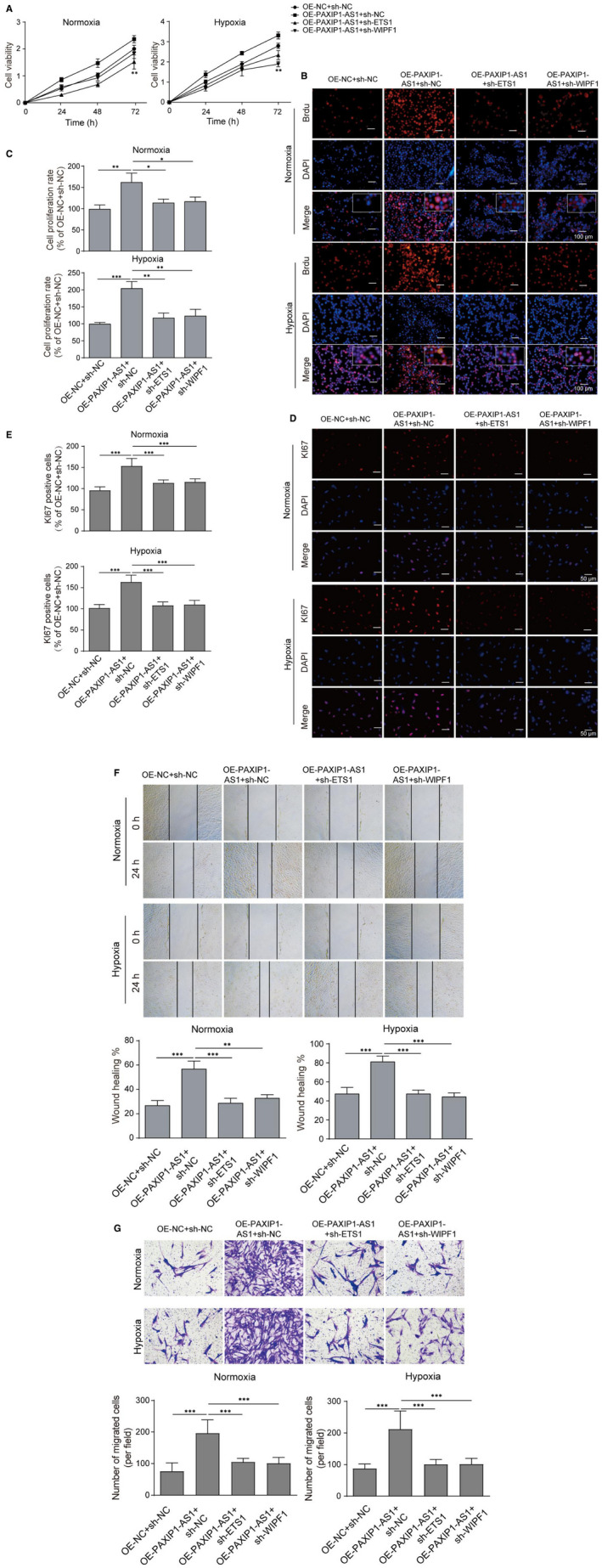
PAXIP1‐AS1 promoted cell viability and migration of hPASMCs through ETS1/WIPF1 signalling. (A) Cell viability was determined by CCK‐8 assay. (B‐E) BrdU assay and immunofluorescence of Ki67 were subjected to validate the cell proliferation in hPASMCs transfected with oe‐PAXIP1‐AS1, combined with sh‐ETS1 or sh‐WIPF1. (F and G) Cell migration was measured by wound healing and transwell assays in hPASMCs transfected with oe‐PAXIP1‐AS1, combined with sh‐ETS1 or sh‐WIPF1. Data are presented as the means ± SD of three independent experiments (n = 3). *P < .05, **P < .01 and ***P < .001

Then, PAXIP1‐AS1, ETS1 and WIPF1 were co‐overexpressed or suppressed and cell viability and migration were measured. As shown in **Figure** **4A**, it was found that the hypoxia condition remarkably enhanced cell viability of hPASMCs. Overexpression of PAXIP1‐AS1 markedly promoted cell viability, while either inhibition of ETS1 or WIPF1 reversed the effects under both in hypoxia and in normal conditions. The data of BrdU assay and immunofluorescence of Ki67 exerted the similar results (**Figure** **4B‐E**). Consistently, wound healing assay also presented that overexpression of PAXIP1‐AS1 apparently facilitated the cell migrative ability, while either inhibition of ETS1 or WIPF1 reversed the positive effects (**Figure** **4F**), which was also confirmed by transwell assay (**Figure** **4G**). All these data indicated PAXIP1‐AS1 promoted cell viability and migration of hPASMCs through regulation of ETS1/WIPF1 axis.

### 3.5 WIPF1 interacted and regulated RhoA

**FIGURE 5:**
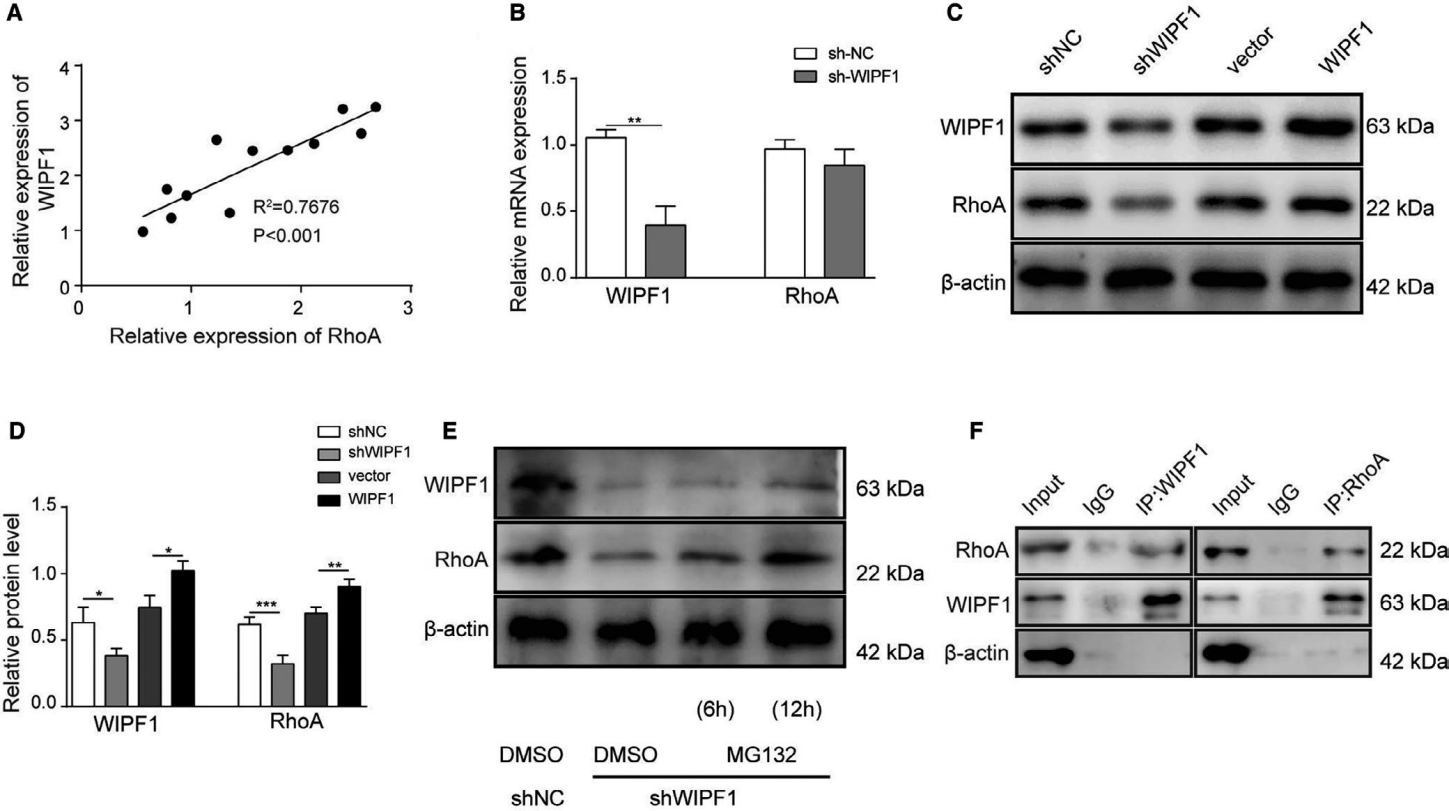
The interaction of WIPF1/RhoA. (A) Pearson analysis was conducted for correlation between WIPF1 and RhoA. (B) mRNA expression of RhoA was determined in cells transfected with sh‐WIPF1 by qRT‐PCR. (C and D) Protein levels of RhoA were determined in cells transfected with sh‐WIPF1 by Western blotting. (E) Protein levels of RhoA were determined in cells transfected with sh‐WIPF1 when MG132 was applied by Western blotting. (F) Co‐IP assay was conducted to confirm the interaction between WIPF1 and RhoA. Data are presented as the means ± SD of three independent experiments (n = 3). *P < .05, **P < .01 and ***P < .001

To further investigate the mechanism of WIPF1 in PAH, the relationship between WIPF1 and RhoA was determined. Results found that the expression of WIPF1 and RhoA was positively correlated in PAH rat model by Pearson's analysis (**Figure** **5A**). When WIPF1 was knocked down, the expression of WIPF1 was remarkably down‐regulated; however, no significant difference was found in mRNA levels of RhoA (**Figure** **5B**). Interestingly, WIPF1 suppression induced remarkable down‐regulation of the protein levels of RhoA (**Figure** **5C,D**), indicating that WIPF1 regulated the post‐translational level of RhoA. It was also found that the decrease of RhoA by suppression of WIPF1 was rescued by proteasome inhibitor MG132 (**Figure** **5E**), indicating that RhoA undergoes proteasomal degradation in the absence of WIPF1. Additionally, Co‐IP analysis demonstrated that WIPF1 could interact with RhoA (**Figure** **5F**).

### 3.6 PAXIP1‐AS1 promoted the pulmonary vascular remodelling via ETS1/WIPF1/RhoA axis in vivo

**FIGURE 6:**
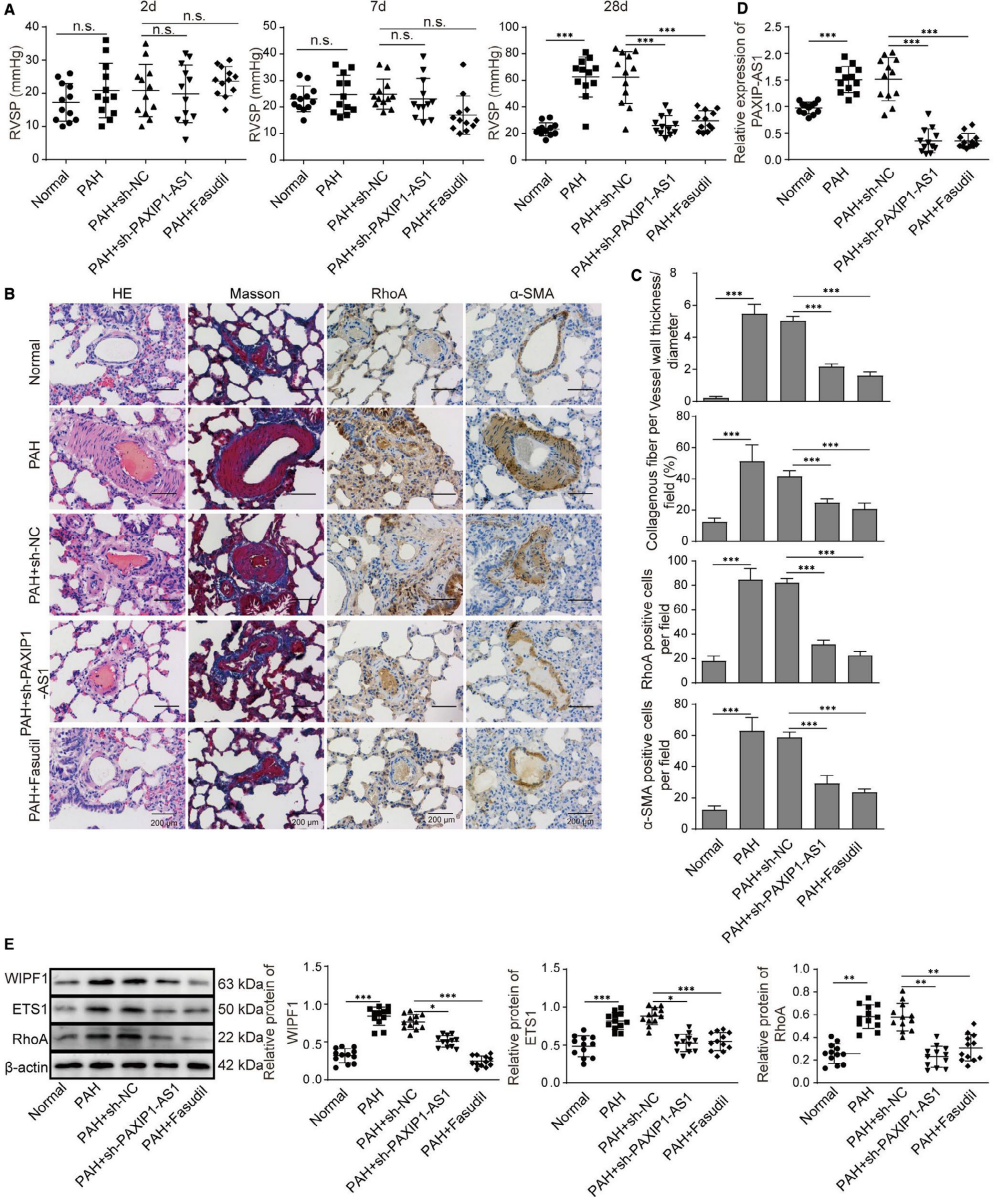
PAXIP1‐AS1 promoted the pulmonary vascular remodelling via ETS1/WIPF1/RhoA axis in vivo. (A) Right ventricle system pressure (RVSP) of healthy rats and PAH rats with/without fasudil or PAH administrated with plasmids expressing sh‐PAXIP1‐AS1. (B and C) H&E staining and Masson's trichrome staining, as well as IHC of RhoA and ɑ‐SMA, were conducted for MCT‐induced lung tissues. Scale bar, 200 μm. (D) Expression of PAXIP1‐AS1 in healthy rats, PAH rats with/without fasudil or PAH administrated with plasmids expressing sh‐PAXIP1‐AS1. (E) Levels of RhoA, ETS1 and WIPF1 in lung tissues from healthy rats and PAH rats with/without fasudil or PAH administrated with plasmids expressing sh‐PAXIP1‐AS1 were determined with Western blotting. Data are presented as the means ± SD of three independent experiments (n = 3). *P < .05, **P < .01 and ***P < .001

Finally, to validate the role of PAXIP1‐AS1 in PAH modulation, lentivirus plasmids expressing sh‐PAXIP1‐AS1 were applied by tail vein injection, and fasudil, a RohA inhibitor, was also used to treat PAH model for confirmation. The RVSP in PAH rat model showed that either treatment of fasudil or inhibition of PAXIP1‐AS1 dramatically reduced the high RVSP in PAH model group (**Figure** **6A**). Similarly, histological examinations of H&E and Masson staining also presented the protective effects of fasudil and sh‐PAXIP1‐AS1 on PAH progression (**Figure** **6B,C**). Besides, IHC staining verified both fasudil and sh‐PAXIP1‐AS1 reduced the expression of RhoA and ɑ‐SMA in PAH lung tissues. The expression of PAXIP1‐AS1 was significantly up‐regulated in PAH model; however, sh‐PAXIP1‐AS1 remarkably down‐regulated its expression, which was also decreased by fasudil (**Figure** **6D**). Western blot analyses also implied that the PAH‐induced high expression of WIPF1, ETS1 and RhoA was inhibited by suppression of PAXIP1‐AS1, and inhibition of RhoA by fasudil also led to inhibition of the expression of WIPF and ETS1 (**Figure** **6E**). Altogether, these findings indicated that PAXIP1‐AS1 promoted the pulmonary vascular remodelling via ETS1/WIPF1/RhoA axis in rat model.

## 4 DISCUSSION

PAH, which has a reported incidence of 1.1‐17.6 per million adults each year, is a rare disorder with high mortality rate.^29^ The aetiology of PAH is not yet fully understood. Several possible pathogeneses have been proposed to uncover the PAH, of which the most prominent feature is the persistent pulmonary resistance. Recently, several advances have been reported in the treatment of PAH. Unfortunately, there remains no pharmacological cure for blocking the PAH progression, due to the complex molecular mechanism and pathogenesis of PAH, involving various intricate pathways, such as impaired NO pathway, aberrant activity of endothelin‐1 pathway.^30^ Thus, it is urgent to develop new PAH drugs, as well as to illustrate deeper insights for molecular mechanisms of PAH. In the present study, we demonstrated for the first time that lncRNA PAXIP1‐AS1 promoted PAH through targeting ETS1/WIPF1 via up‐regulating RhoA.

PAXIP1‐AS1 is a newly identified lncRNA with limited studies on its biofunctions. In this study, we firstly observed that PAXIP1‐AS1 was elevated in PAH animals. In previous researches, it has been demonstrated that PAXIP1‐AS1 was highly abundant and might be associated with the hyperproliferative and migratory actions of idiopathic PAH smooth muscle cells and could interfered with the focal adhesion axis via regulation of paxillin.^18^ This study is consistent with our result. Another study also revealed that PAXIP1‐AS1 promoted cell invasion and angiogenesis of glioma by recruiting transcription factor ETS1.^22^ Despite these studies, there is no study exactly explaining the molecular mechanisms of PAXIP1‐AS1 in PAH. In the present study, we demonstrated for the first time that PAXIP1‐AS1 promoted cell viability and migration of hypoxia‐induced hPASMCs through ETS1/WIPF1/RhoA axis.

To further investigate the molecular mechanism of PAXIP1‐AS1 in PAH, the relationship between PAXIP1‐AS1 and ETS1 was confirmed, and we also demonstrated that the levels of ETS1 were increased in PAH rats. Actually, the relationship between ETS1 and PAXIP1‐AS1 has been previously observed in the above study in glioma.^22^ Besides, ETS1 was also found to promote proinflammatory responses and neointima formation in carotid artery endoluminal vascular injury.^31^ In lung diseases, ETS1 acts as an oncogene and could promote cell viability and migration of lung cancer cells.^26^ However, no study reported the role of ETS1 in the pathogenesis of PAH. In this study, we investigated that ETS1 was up‐regulated in PAH and could also promote cell viability and migration of hypoxia‐induced hPASMCs by regulating WIPF1/RhoA signalling. And PAXIP1‐AS1 could recruit transcription factor ETS1 to influence cell function of hPASMCs in PAH.

Next, the regulatory effects of ETS1 on WIPF1 were also observed and the axis of PAXIP1‐AS1/ETS1/WIPF1 was established. WIPF1 has been widely reported in cancer development. It was found up‐regulated WIPF1 played a key role in BRAF V600E‐promoted papillary thyroid cancer aggressiveness.^32^ Pan et al reported WIPF1 reversed the anti‐cancer effects of miR‐141/200c in pancreatic ductal adenocarcinoma.^33^ Interestingly, WIPF1 was found as a downstream target of ETS1 in lung cancer.^26^ To our best of knowledge, up to now, no study reported role of WIPF1 in PAH or hypertension. Here, we presented that WIPF1 was positively correlated with ETS1 and was regulated by PAXIP1‐AS1/ETS axis in PAH. The inhibition of WIPF1 reversed the promotion effects of PAXIP1‐AS1 on PAH.

Finally, the role of RhoA in PAH and its relationship with the PAXIP1‐AS1/ETS1/WIPF1 axis were demonstrated. Rho‐associated protein kinases (ROCK subtypes 1 and 2) are commonly activated by RhoA, which participates in various physiological processes, such as cell migration, cell contraction and proliferation. Previous studies demonstrated that the activity of RhoA and Rho kinase was twofold in PAH patients compared to the healthy subjects.^12^ RhoA/ROCK axis was demonstrated to be involved in the PAH pathogenesis through modulating vasoconstriction and vascular remodelling, the main pathogenic events of PAH.^34^ Moreover, Yu and the colleagues reported that in heparin‐treated PASMCs, GEF, RhoA and ROCK were increased, leading to the down‐regulated of p27, which contributed to the PASMC proliferation.^35^ In the present research, we also found that RhoA was activated in PAH models and we firstly found RhoA could be activated by WIPF1. Fasudil, a RhoA‐ROCK inhibitor, is commonly used as a vasodilator in cerebral vasospasm, which is also seen as a therapeutic agent in PAH treatment.^36^ Fasudil is reported to inhibit RhoA signalling in many diseases such as cardiomyopathy and osteosarcoma.^37^, ^38^ A single dose of Rho kinase inhibitor fasudil was reported to remarkedly reduce the mean pulmonary arterial pressure and vascular resistance in a sample of nine patients with severe PAH.^39^ Similarly, our study also confirmed that RhoA was obviously up‐regulated in lung tissues with PAH, contributing to PASMC proliferation, while the application of fasudil or inhibition of PAXIP1‐AS1 attenuated these effects. These results are consistent with the above studies. Besides, we also found RhoA was a downstream target of WIPF1, which was also observed in a previous research in cancer.^40^

The present study also has some limitations. Firstly, deeply molecular mechanism of how PAXIP1 was regulated is still unclear. Secondly, clinical significance of PAXIP1‐AS1 should be further confirmed in PAH patients. All these need more studies to illustrate.

To conclude, our study revealed a novel mechanism by which PAXIP1‐AS1/ETS1/WIPF1/RhoA axis regulated the development of PAH, helping better understanding the pathology of PAH and further validating PAXIP1‐AS1 as a therapeutic target for PAH treatment.

## CONFLICT OF INTEREST

The authors declare that there is no conflict of interest.

## AUTHOR CONTRIBUTION

**Rong Song:** Conceptualization (equal). **Si Lei:** Methodology (equal). **Song Yang:** Data curation (equal). **Shang‐Jie Wu:** Writing‐original draft (equal); Writing‐review & editing (equal).

## ETHICAL APPROVAL

All animal care and experimental procedures were approved by the Animal Care Ethics and Use Committee of the Second Xiangya Hospital of Central South University and performed in accordance with the guidelines of this Committee.

## CONSENT FOR PUBLICATION

Not applicable. This article does not contain any studies with human participants performed by any of the authors.

## Data Availability

All data generated or analysed during this study are included in this article. The data sets used and/or analysed during the current study are available from the corresponding author on reasonable request.
